# Mediterranean Diet and Cardiovascular Disease: The Moderating Role of Adequate Sleep—Results from the ATTICA Cohort Study (2002–2022)

**DOI:** 10.3390/nu16010012

**Published:** 2023-12-20

**Authors:** Evangelia Damigou, Christina Chrysohoou, Christina Vafia, Fotios Barkas, Evrydiki Kravvariti, Elpiniki Vlachopoulou, Konstantina Kyrili, Costas Tsioufis, Christos Pitsavos, Evangelos Liberopoulos, Petros P. Sfikakis, Demosthenes Panagiotakos

**Affiliations:** 1 Department of Nutrition and Dietetics, School of Health Sciences and Education, Harokopio University, 17676 Athens, Greece; edamigou@hua.gr (E.D.);; 2 First Cardiology Clinic, Medical School, National and Kapodistrian University of Athens, Hippokration Hospital, 15772 Athens, Greece; 3 Department of Internal Medicine, Medical School, University of Ioannina, 45500 Ioannina, Greece; 4 First Department of Propaedeutic Internal Medicine, Medical School, National and Kapodistrian University of Athens, Laiko General Hospital, 15772 Athens, Greece

**Keywords:** sleep, adequate sleep, Mediterranean diet, diet, cardiovascular disease, Mediterranean dietary pattern

## Abstract

The relationship between diet, sleep duration and cardiovascular disease (CVD) has not been well understood. The aim of the present study was to test the potential modifying role of sleep duration in the association between adherence to the Mediterranean-type diet (MD) and CVD risk. The study consisted of *n* = 313 initially free-of-CVD adults, from the ATTICA cohort study (2002–2022), with available information on sleep habits. Sleep habits were categorized as inadequate and adequate sleep duration (< or ≥7 h/day, respectively). In multi-adjusted analysis, MD adherence was inversely associated with CVD risk [Hazard Ratio-HR per 1/55 in MedDietScore: 0.80, 95% Confidence Interval-CI: 0.65, 0.98]. A significant interaction between sleep duration and MedDietScore was observed (*p* < 0.001). In subgroup analysis, the protective association between MD adherence and CVD risk was found only in participants who slept adequately, i.e., >7 h/day [HR:0.80, 95%CI: 0.65, 0.98]. Those who had a high adherence to the MD along with adequate sleep habits, had a 70% reduced 20-year CVD risk [HR:0.30, 95%CI: 0.11, 0.80], compared to those who had a low MD adherence and inadequate sleep habits. Sleep duration should be a part of an individual’s lifestyle, together with dietary and other habits, to effectively evaluate CVD risk for future events.

## 1. Introduction

The relationship between cardiovascular disease (CVD) and the Mediterranean type diet (MD) has been well documented and established in the literature, and has been of interest for the last 60 years [1,2]. Organizations both inside and outside the Mediterranean region, such as the European Society of Cardiology (ESC) and the American Heart Association/American College of Cardiology (AHA/ACC), recommend following a MD or similar diet to prevent the development of CVD [3,4], despite the current decline in the MD adherence that has been observed worldwide [5,6,7,8,9].

The relationship between sleep and health has also gained interest these past years, and has reached a peak these last two decades [10]. Depending on an individual’s age and lifestyle, sleep habits may vary, but it is estimated that humans spend approximately 1/3 of their lives sleeping [10]. Research suggests that sleep is an essential body function that has multiple benefits for health and especially cardio-metabolic health [10,11,12,13,14]. Specifically, both short and long sleep duration has been positively associated with increased CVD risk, in systematic reviews and meta-analyses [11,14]. Makarem et al. (2022) studied objectively the sleep habits (i.e., evaluated via overnight polysomnography, 7-day wrist actigraphy, and validated questionnaires) of 1920 participants from the multi-ethnic study of atherosclerosis (MESA), and found that the addition of sleep, in cardiovascular health scores (defined and computed based on AHA’s “Simple 7”), improved CVD outcome prediction [12]. Afterwards, sleep was acknowledged as an important lifestyle characteristic for CVD prevention by AHA; sleep was included in AHA’s recommended components for acquiring cardiovascular health and diminishing CVD risk, changing AHA’s “Simple 7”, to the newly adopted “Life’s essential 8” [15,16].

Few studies have associated the adherence to the MD with better sleep habits. It has been proposed that this positive association might be explained through the better management of body weight, via the adoption of MD [17,18,19]. Sleep disorders such as sleep apnea have been associated with increased CVD risk [20]. To the best of our knowledge, the relation among MD, sleep and CVD risk has been studied in only one previous study, by Wang et al., in which they evaluated 23,212 individuals from the National Health and Nutrition Examination Survey (NHANES), and found that lower adherence to a MD in the presence of sleep disorders synergistically increase all-cause and CVD mortality [21]. However, it should be clarified that this study examined only sleep disorders, and not normal sleep habits, in relation to mortality (i.e., all-cause and CVD mortality).

Thus, as the relationship among sleep duration, MD adherence and CVD risk (especially non-fatal CVD events) has not been adequately elucidated, the aim of the present study was to evaluate whether sleep duration could act as a moderator in the relationship between the adherence to the MD and CVD risk (fatal/non-fatal CVD events) in a Mediterranean population.

## 2. Materials and Methods

### 2.1. Study Design

The ATTICA study is a prospective cohort study, which started in 2001–2002 (baseline) and afterwards had 3 follow-ups at 5, 10, and 20 years (i.e., 2006, 2012, 2022). The study included 3042 adult men and women, from the Attica region, in Greece (including the capital, Athens). The study aimed to assess socio-demographic, clinical, lifestyle, biochemical, and psychological parameters related to CVD, and to explore the associations between the aforementioned factors and long-term CVD incidence. Participants’ characteristics were assessed through face-to-face interviews with the study’s investigators. Details about the study can be found in previously published papers [22,23,24,25].

### 2.2. Bioethics

The study was carried out in accordance with the Declaration of Helsinki (1989) of the World Medical Association and was approved by the Institutional Ethics committee of Athens Medical School (#017/1.5.2001). All participants were informed about the study aims and provided written consent to participate in the study.

### 2.3. Baseline and Follow-Up Measurements

#### 2.3.1. Demographic Characteristics

Demographic characteristics included age, sex, and socio-economic status (SES), which was assessed as a combined measure of mean annual income and education. Participants were categorized as of low SES (i.e., those who had up to 9 years of schooling and low/medium income or up to 14 years of schooling and low income), of high SES (i.e., those who had 15 or more years of schooling and high income or 10–14 years of schooling and very high income) and of middle SES (i.e., the rest of the participants).

#### 2.3.2. Clinical Characteristics

Clinical characteristics, CVD, hypertension, hypercholesterolemia and type 2 diabetes mellitus, were assessed similarly in all evaluations, by study investigators, in a clinical setting, and defined according to WHO-ICD-10 [22].

Briefly, at baseline, biochemical characteristics (glucose and lipids) were measured in 12-h fasting blood samples using chromatographic enzymic method in a Technicon automatic analyzer RA-1000 (Dade Behring, Marburg, Germany) [22]. For the measurements, serum was harvested immediately after admission.

Hypercholesterolemia was defined as TC ≥ 200 mg/dL and/or use of hypocholesterolemic agents [26]. Type 2 diabetes mellitus was based on fasting plasma glucose ≥126 mg/dL or use of antidiabetic drugs [27]. Participants’ arterial blood pressure was measured based on three measurements after a >30 min rest period and while in a sitting position, using a manometric device (ELKA aneroid manometric sphygmometer, Von Schlieben Co., Munich, Germany). Hypertension was defined as an average systolic blood pressure/diastolic blood pressure exceeding 140/90 mmHg or use of antihypertensive drugs [28].

#### 2.3.3. Anthropometric Characteristics

Body weight and height were measured according to standard procedures. Body mass index (BMI) was computed as weight/height^2^ (kg/m^2^). According to BMI measurements at 2002 and 2012, participants’ body weight status were categorized as follows: (i) never overweight/obese: those who had a BMI < 25 kg/m^2^ at both evaluations, (ii) became overweight/obese: those who had a BMI < 25 at 2002, but ≥25 kg/m^2^ in 2012, (iii) became non-overweight/obese: those who had a BMI ≥ 25 in 2002, but <25 kg/m^2^ in 2012, and (iv) always overweight/obese: those who had a BMI ≥ 25 kg/m^2^ at both evaluations.

#### 2.3.4. Lifestyle Habits

Smoking trajectories were evaluated in the 2002 and 2012 examinations, and participants were categorized into (i) never smoked: those who did not smoke at both evaluations, (ii) started smoking: those who did not smoke in 2002, but smoked in 2012, (iii) stopped smoking: those who smoked in 2002, but did not smoke in 2012, (iv) always smoked: those who smoked at both evaluations. According to their physical activity trajectories (2002–2012), evaluated by the International Physical Activity Questionnaire, participants were categorized as follows: (i) always physically inactive: those who did not engage in physical activity at both evaluations, (ii) started physical activity: those who did not engage in physical activity in 2002, but started being physically active in 2012, (iii) stopped physical activity: those who engaged in physical activity in 2002, but did not in 2012, and (iv) always physically active: those who engaged in physical activity at both evaluations.

#### 2.3.5. Dietary Habits

Participants’ dietary habits were assessed through a validated for the Greek population Food Frequency Questionnaire (FFQ) [29]. Adherence to the MD was evaluated through MedDietScore (range: 1–55, with higher values signifying higher adherence) [30]. MedDietScore has 11 components: 7 typical Mediterranean foods/food groups (i.e., fruits, vegetables, whole grains, potatoes, legumes, fish, and olive oil) which are scored on a positive scale (0–5, for very rare to very frequent consumption), 3 non-Mediterranean foods/food groups (full-fat dairy products, poultry, and red meat) which are scored on the opposite scale, and alcohol which is scored on a non-linear scale (0 for consumption of 0 and >7 servings/day, 1 to 5 for consumption of 6–7, 5–6, 4–5, 3–4, and 1–3 servings/day, respectively). Based on participants’ adherence to the MD, 4 trajectories were formed: (i) always close to the MD: median MedDietScore in 2002 and 2012: ≥27, (ii) from close to away: median MedDietScore score in 2002: ≥27, in 2012: <27, (iii) from away to close: median MedDietScore in 2002: <27, in 2012: ≥27, (iv) always away: median MedDietScore value in 2002 and 2012: <27.

#### 2.3.6. Sleep Habits

Sleep habits assessment was conducted in the 2012 examination of the participants. For the purposes of the current study, data on sleep duration (i.e., sleep hours per day) during the past 4 weeks were assessed. Participants were categorized as having inadequate sleep (i.e., those who slept <7 h/day) or adequate sleep (i.e., those who slept ≥7 h/day) [31].

### 2.4. Study Sample

**Figure 1:**
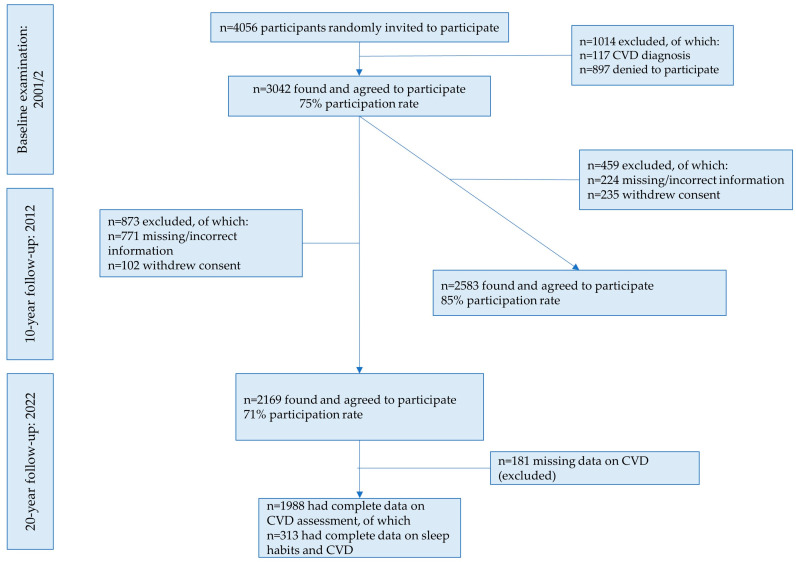
Flow-chart of the study sample during 2002–2022 (*n* = 313). Abbreviations: CVD: cardiovascular disease.

From the 3042 initially free-of-CVD participants, 2169 were found at the 20-year follow-up. Of them 1988 had complete data on CVD. For details on the ATTICA study participants during 2002–2022 please see Figure 1. However, for the present study, we used a sub-sample of the ATTICA study participants, who had completed a questionnaire on sleep habits during the 10-year follow-up, in 2012 (*n* = 377). Thus, the sample of the current study was 313 participants who were found at the 20-year follow-up and had complete data on CVD assessment. Differences in the age and sex distribution between this sample and the initial one was not observed (*p*-value < 0.05).

This sample was sufficient to obtain a statistical power of 80% or higher to evaluate two-sided statistical hypotheses of hazard ratios of CVD of 1.30 or higher, 0.05 type-I error rate.

### 2.5. Statistical Analysis

For the statistical analyses, STATA version 17 (STATA Corp, College Station, TX, USA) was used. Categorical variables are presented as frequencies, while continuous variables as mean values ± standard deviation for normally distributed variables (i.e., age) or as median (interquartile range—IQR) for non-normally distributed variables (i.e., MedDietScore at 2002 and 2012). Continuous variables were tested for normality through probability–probability (P-P) plots. Associations between categorical variables were tested using the chi-squared test. After controlling for equality of variances using Levene’s test, comparisons of mean values of normally distributed variables (i.e., age) between CVD and CVD-free participants or between participants with adequate or inadequate sleep habits were performed using Student’s *t*-test. Comparisons of mean values of non-normally distributed variables (i.e., MedDietScore at 2002 and 2012) between CVD and CVD-free participants or between participants with adequate or inadequate sleep were performed using the Mann–Whitney non-parametric test. Among the four groups of concomitant MD adherence and sleep habits (i.e., low MD adherence and inadequate sleep, low MD and adequate sleep, high MD and inadequate sleep, high MD and adequate sleep), comparisons of mean values of normally distributed variables (i.e., age) were performed using one way analysis of variance (ANOVA), while for non-normally distributed ones (i.e., MedDietScore at 2002 and 2012), the Kruskal–Wallis non-parametric test was used. *p*-values from post-hoc comparisons were adjusted using the Bonferroni rule. Hazard ratios (HR) and their corresponding 95% confidence intervals (95% CIs) for MedDietScore in relation to CVD within the 20-year period were taken from multivariable Cox-regression analysis. Multiple hazards models were used to progressively adjust for potential confounding factors. Models used included Model 1: crude model, Model 2: adjusted for age, sex and SES, Model 3: Model 2 and further adjustment for hypertension, hypercholesterolemia, diabetes, family history of CVD, Model 4: Model 3+ trajectories of body weight, physical activity level, MD adherence, smoking habits, and Model 5: Model 4+ adequate sleep. Time to CVD event was recorded on annual basis. Participants with missing values were excluded from the analysis. A significant interaction was observed between MedDietScore and sleep habits; therefore, a subgroup analysis was performed in order to evaluate the relationship between MedDietScore and 20-year CVD risk, according to sleep habits (adequate and inadequate sleep). Additionally, the classification and regression tree (CART) analysis was used to determine the most significant factors between CVD incidence and several contributing factors, including MD adherence, sleep habits, age, and sex [32]. Lastly, multiple hazards models (models 1–4), were also used to evaluate the relationship between the concomitant adherence to the MD and sleep habits, with CVD risk.

## 3. Results

During the 20-year follow-up, approximately 31.6% of the participants (*n* = 99) developed a CVD event. Participants slept a total of mean (standard deviation) 6.3 (1.2) hours per day. The majority of the participants (i.e., 57.5%) reported inadequate sleep habits (i.e., slept < 7 h/day); at this point it should be noted that sleeping hours refer to total hours per day, but mid-day nap was not reported in the studied sample.

### 3.1. Baseline and Longitudinal Participants’ Characteristics by CVD Status at 20-Year Follow-Up

Participants’ characteristics, both at baseline and longitudinally, by cardiovascular disease status in the 20-year follow-up are presented in Table 1. Participants who developed CVD during the 20 years, compared to participants who did not, were mainly older participants, and a higher percentage of them had a history of hypertension, hypercholesterolemia, and diabetes mellitus both at baseline and in the follow-up. Moreover, a lower percentage of the participants slept adequately (in 2012), and a higher percentage started smoking (in 2012), were overweight or obese, physically inactive, and away from the MD (during 2002–2012) (Table 1).

### 3.2. Baseline and Longitudinal Participants’ Characteristics by Sleep Habits

Participants’ characteristics by sleep habits at the 10-year follow-up are shown in Table 2. Participants who had adequate sleep (≥7 h/day), compared to participants who had inadequate sleep (<7 h/day), were younger, had a higher SES, a higher history of hypertension (at baseline and in 2022), a higher history of hypercholesterolemia (in 2022), and a higher MedDietScore (at baseline and in 2012) (Table 2).

### 3.3. Baseline and Longitudinal Participants’ Characteristics by Concomitant Adherence to the Mediterranean Diet and Sleep Habits

Participants’ characteristics by concomitant adherence to the MD and sleep habits at the 10-year follow-up are shown in Table 3. Between the four groups (low MD adherence and inadequate sleep, low MD and adequate sleep, high MD and inadequate sleep, and high MD and adequate sleep), differences were observed concerning age, sex, SES, history of clinical characteristics, trajectories of body weight, and MD adherence. In the high MD adherence and adequate sleep category, compared to the low MD and inadequate sleep category, participants were more likely to be younger, women, of a high SES, were less likely to have a history of clinical conditions, and had (initially or continuously) a higher adherence to the MD and a normal weight (Table 3). Those in the high MD and inadequate sleep group, compared to those in the low MD and adequate sleep, were more likely to be younger, women, of middle SES, and to have a better weight status in the long-term (Table 3).

### 3.4. Mediterranean Diet and 20-Year CVD Incidence

Results from Cox proportional hazards models, investigating the association between the MD and 20-year CVD incidence, are presented in Table 4. In Models 1–4, the adherence to the MD, per 1/55 increase of MedDietScore was inversely associated with a reduction on CVD risk, varying from 15% in the crude model (Model 1) to 9% in the fully adjusted model (Model 4).

### 3.5. The Moderating Role of Adequate Sleep in the Association between MD Adherence and CVD Incidence

The addition of adequate sleep in Model 5, modified the association between adherence to the MD and CVD, making it insignificant (Table 4, Model 5). Afterwards, subgroup analyses were performed (p-for-interaction between MedDietScore and sleep duration <0.001), concerning the association between CVD risk and MD adherence according to sleep habits (adequate and inadequate sleep) (Table 5). It was found that sleep habits moderated the protective effects of the MD against CVD risk, as MedDietScore was inversely associated with CVD risk only in participants who slept adequately (≥7 h/day) (Table 5).

**Figure 2:**
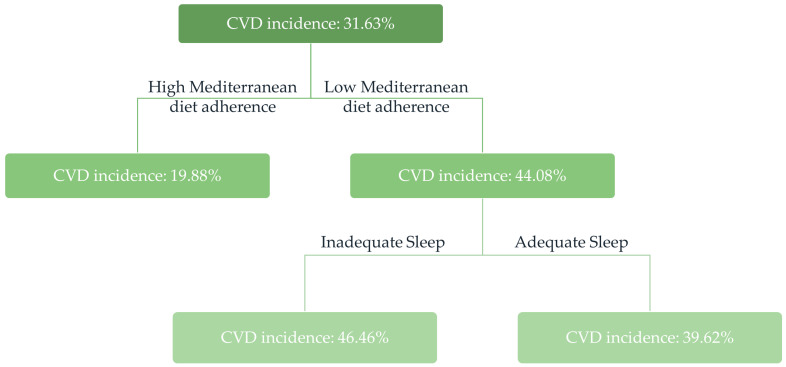
Classification tree model studying CVD incidence in relation to MD adherence, sleep habits, and age and sex in the ATTICA study sample during 2002–2022 (*n* = 313). Adequate sleep is defined as ≥7 h/day, and inadequate sleep as <7 h/day. Low MD adherence was defined as MedDietScore during 2012 <27 (median), and high MD adherence as MedDietScore during 2012 ≥27 (median). Abbreviations: CVD: cardiovascular disease, MD: Mediterranean-type diet.

For a more straightforward and clinically relevant depiction, Figure 2 presents the classification tree concerning CVD incidence in relation to MD adherence, sleep habits, and age and sex; no differences by age and sex were observed.

### 3.6. Mediterranean Diet Adherence and Sleep Habits in Relation to CVD Risk

Table 6 depicts results from Cox proportional hazards models, evaluating the association between the adherence to the MD and sleep habits in relation to 20-year CVD incidence. In the crude model, both high adherence to the MD and inadequate or adequate sleep was inversely associated with 20-year CVD risk. However, in the fully adjusted model (Model 4), only those who had a high adherence to the MD along with adequate sleep habits had a 70% reduced risk to develop CVD during the 20 years of follow-up, compared to those who had a low MD adherence and inadequate sleep habits (Table 6).

## 4. Discussion

This study aimed to evaluate the potential modifying role of sleep duration in the relationship between the adherence to the MD and CVD risk. It was found that sleep duration (hours sleeping/day) modifies this relationship, and that, in specific, adherence to the MD was protective against CVD risk, in multi-adjusted models, only in participants who had adequate sleep habits (i.e., those who were sleeping ≥ 7 h/day), but not in those who had inadequate sleep habits (i.e., those who were sleeping < 7 h/day). Moreover, those who reported a high adherence to the MD along with adequate sleep habits, had a 70% reduced 20-year risk to develop CVD [HR: 0.30, 95%CI: 0.11, 0.80], compared to those who had a low MD adherence and inadequate sleep habits. Despite the limitations this cohort study may carry, to the best of our knowledge, this is the first study to propose a moderating role of sleep in the well-established protective effect of the MD against CVD, therefore suggesting that an individual’s overall lifestyle, sleep habits included, should be addressed to effectively reduce CVD risk, as it has also been suggested recently by the American Heart Association [16].

### 4.1. Sleep Duration and Cardiovascular Disease

Sleep duration has shown a U- or J-shaped relationship with CVD; both short sleep duration (i.e., usually less than 5–6 h/night), as well as long sleep duration (i.e., usually more than 7–8 h/night) have been associated with an increased CVD risk [10,11,14,33,34]. It has been observed that approximately 33% of adults in the US reported inadequate sleep habits in 2017 (i.e., slept less than 6 h/night) [35]. In our study, an even higher percentage (58%) compared to the US adults, had inadequate sleep (i.e., <7 h/day) in 2012, potentially attributed to secular lifestyle trends such as having multiple jobs, working shifts or irregular hours [10,36]. Moreover, our study sample slept less compared to another sample of Greek adults, from the Hellenic National Nutrition and Health Survey (HNNHS) (i.e., mean hours sleeping: 6.3 vs. 7 h/night) [37]; this could be attributed to the fact that our study had older participants, as sleep habits change during the aging process, independently of other factors [38].

It should be noted that, in this study, we used the threshold of 7 h of sleep duration per day. This was based on systematic reviews and meta-analyses, which have revealed that sleeping less than 6 h/day or more than 8–9 h/day, is positively associated with increased CVD risk, compared to 6 to 8 h/day [10,11,14,33]. Moreover, the American Academy of Sleep Medicine and Sleep Research Society recommend that adults should sleep more than 7 h per day for optimal health, and the American Heart Association recommends sleeping 7–9 h/night to prevent CVD [16,39]. In addition, we only categorized sleep as adequate or inadequate, because only a small percentage (i.e., 1.6%) slept more than 9 h/day. In other studies, regarding long sleep duration, it has been proposed that depression and low SES are factors that might influence the relationship between long sleep duration and increased mortality [40].

Moreover, mid-day nap was not reported in the studied sample. In addition, instead of total sleep duration, most studies evaluate the association between midday-naps or siestas (i.e., day-time naps, usually taken in the early afternoon and/or after the midday meal in the Mediterranean lifestyle) and health [41,42,43,44]. Daytime naps have shown mixed results in the literature concerning mortality and CVD risk, depending on the reason people have daytime naps; if the reason is for stress-relief, as it was common in the Mediterranean lifestyle, daytime naps might have beneficial effects, but if the reason is to fix inadequate night sleep due to symptoms such as depression or cognitive decline, daytime naps might not suffice to reverse the increased CVD risk associated with these diseases [10,41,42]. Thus, future studies on the effect of daytime naps on CVD risk should consider overall sleep habits and either use sleep duration as a covariate or differentiate results by sleep duration.

### 4.2. The Importance of Sleep for Cardiovascular Health

Sleep helps the body and mind to function and perform well during the day; sleep could be considered as the best thing one could do by doing “nothing”. Most recently, the World Sleep Society Global Sleep Health Taskforce emphasized the need to integrate sleep in public health campaigns [45]. Moreover, short sleep might modulate the association between established risk factors (such as hypertension, diabetes mellitus, dyslipidemia) and CVD [46,47]. Chronic inadequate sleep may lead to electrocardiographic abnormalities, enhanced release of norepinephrine, inhibited muscle sympathetic nerve activity, enhanced cardiac sympathetic drive, deterioration of vascular structure and function, arterial stiffness, and increased oxidative stress levels as well as increases in blood pressure, glucose dysregulation, decreased insulin sensitivity, weight gain, and disrupted circadian rhythm [36,48,49,50].

### 4.3. Diet, Sleep, and Cardiovascular Disease

Sleep and diet might reciprocally and multi-directionally affect CVD risk [51,52]. Studies have shown that short sleep duration or lower sleep quality is associated with lower diet quality and adherence to MD [52,53,54,55] and that better dietary habits and greater adherence to MD might be associated with better sleep habits [51,55,56]. The increase in the availability of tryptophan (a precursor of serotonin and melatonin) and its uptake by the brain, after consumption of carbohydrates and especially fruits, vegetables and wholegrains, that are abundant and usually contribute the largest proportion of energy intake in the Mediterranean dietary pattern, have been associated with better sleep habits (i.e., decreased sleep latency, time to sleep initiation, and better sleep quality) [51,52,57,58,59,60]. Moreover, the consumption of foods containing tryptophan such as milk, fatty fish, kiwis, and cherries, have been associated with better sleep habits [52].

Sleep loss might adversely influence dietary behaviors, lead to weight gain and an elevated risk for CVD through the dysregulation of ghrelin and leptin as well as the consumption of high-energy or high-fat foods, due to the need to have energy to stay awake, increased time and opportunities to eat, greater sensitivity to food reward, and psychological distress [61]. Additionally, the gut microbiome might also play a role in the relationship between sleep, dietary habits, and CVD risk [51]. Mediterranean type diets are rich in probiotics and prebiotics and following such diets is related to better gut microbiome profiles, while the relationship between sleep and the gut microbiome might be reciprocal; evidence suggests that sleep influences the gut microbiome, and some studies have proposed that the gut microbiome composition might also affect sleep habits [51,62,63].

It should be mentioned that the relationship between the adherence to the MD along with sleep habits and CVD is rarely explored. Recently, in the National Health and Nutrition Examination Survey (NHANES), it was found that lower adherence to a MD and sleep disorders synergistically increased all-cause and CVD mortality [21]. This study seems to be in line with our observation that sleep duration modifies the association between adherence to the MD and CVD risk. Moreover, our study results suggest that the protective effect of MD against CVD is clearly outspoken in those who sleep adequately. The interrelation between diet, sleep and CVD is a relationship that warrants further research, through clinical studies and epidemiological studies with bigger samples.

### 4.4. Strengths and Limitations

The ATTICA study has a long follow-up and multiple assessments, that allowed the inclusion of trajectories of clinical and lifestyle characteristics as covariates in the relationship among sleep, MD, and CVD. However, some limitations should be noted. The ATTICA study was not designed to study specifically sleep habits, as is the case for most epidemiological studies which have reported data on sleep habits. Sleep habits were assessed only once, in 2012 (during the 3rd evaluation), therefore trajectories of sleep habits could not be computed, and changes in sleep habits (e.g., due to different working hours or stress levels) could have affected our results. Moreover, sleep habits were self-reported by the use of a validated questionnaire, provided by study investigators, and not by other direct measurements (e.g., polysomnography), but this is common in epidemiological studies that assess sleep characteristics such as sleep duration or sleep quality. Additionally, we assessed sleep duration as adequate or inadequate sleep (because only a small percentage, i.e., 1.6%, slept more than 9 h/day) and did not take into consideration day-time naps. The relatively small sample size of the current study could be another limitation; men and women were analyzed together, and this fact might have masked potential sex-differences. However, it should also be noted that although the sample was not of a large size, the effects of sleep are clearly outspoken. Furthermore, dietary habits were assessed by an FFQ; although, this FFQ was validated for the Greek population and provided by study investigators, some measurement error could have existed.

## 5. Conclusions

In conclusion, in this study, it was found that sleep duration was a moderator in the well-established relationship between MD adherence and CVD risk. Therefore, sleep should have a place among other lifestyle habits that are recommended in clinical practice as well as public health actions, for an individual to acquire and maintain better cardiovascular health.

## Figures and Tables

**Table 1:** Baseline and longitudinal characteristics by cardiovascular disease status at the 20-year follow-up in the ATTICA study sub-sample (*n* = 313).

	CVD Status at 20-Year Follow-Up		*p*-Value *
Baseline Socio-Demographic Characteristics	No	Yes	
***N***	**214**	**99**	
Age, mean (SD)	39.5 (7)	53 (8)	**<0.001**
Sex, %men	57.5	56.6	0.880
SES, %			0.220
Low	6	6	
Middle	44	56	
High	50	37	
**Baseline clinical characteristics**			
History of hypertension, %	20	41	**<0.001**
History of hypercholesterolemia, %	34	68	**<0.001**
History of diabetes mellitus, %	0.93	8	**0.001**
Family history of CVD, %	49	44	0.549
**Clinical characteristics at 20 years**			
History of hypertension, %	37	72	**<0.001**
History of hypercholesterolemia, %	72	91	**<0.001**
History of diabetes mellitus, %	26	38	**0.026**
**Anthropometric characteristics**			
Body weight trajectories 2002–2012, %			**<0.001**
Never overweight/obese	21	10	
Became overweight	30	15	
Became non-overweight/obese	17	25	
Always overweight/obese	31	50	
**Lifestyle characteristics**			
Smoking trajectories (2002–2012), %			**<0.001**
Never smoked	53	46	
Started smoking	5	23	
Stopped smoking	14	12	
Always smoked	26	18	
Physical activity trajectories (2002–2012), %			**<0.001**
Always physically inactive	34	55	
Started physical activity	21	2	
Stopped physical activity	29	41	
Always physically active	16	2	
Mediterranean diet trajectories (2002–2012), %			**<0.001**
Always close	22	7	
From close to away	55	36	
From away to close	12	26	
Always away	11	30	
MedDietScore (2002), Median (IQR)	27 (2.5)	25 (1.9)	**<0.001**
MedDietScore (2012), Median (IQR)	26 (2.7)	25 (3.1)	**<0.001**
Adequate sleep (≥7 h/day) (2012), % yes	48	30	**0.003**

* *p*-Values referring to differences between CVD status and categorical variables are derived from the chi-squared test. *p*-Values referring to differences between CVD status and continuous variables are derived from Student’s *t*-test (i.e., age, normally distributed) or the Mann–Whitney nonparametric test (i.e., MedDietScore at 2002 and 2012, non-normally distributed). Bold indicates statistical significance. Abbreviations: CVD: cardiovascular disease; IQR: interquartile range, SD: standard deviation; SES: socioeconomic status.

**Table 2:** Baseline and longitudinal characteristics by sleep habits during the 10-year follow-up in the ATTICA study sub-sample (*n* = 313).

	Sleep Habits		*p*-Value *
Baseline Socio-Demographic Characteristics	Inadequate Sleep (<7 h/Day)	Adequate Sleep (≥7 h/Day)	
***Ν***	**180**	**133**	
Age, mean (SD)	45 (10)	41 (10)	**0.001**
Sex, %men	59	54	0.310
SES, %			**0.002**
Low	8	4	
Middle	55	36	
High	37	60	
**Baseline clinical characteristics**			
History of hypertension, %	29	20	**0.050**
History of hypercholesterolemia, %	45	41	0.449
History of diabetes mellitus, %	4.6	1.9	0.151
Family history of CVD, %	46	46	0.506
**Clinical characteristics at 20 years**			
History of hypertension, %	56	38	**0.001**
History of hypercholesterolemia, %	81	71	**0.038**
History of diabetes mellitus, %	32	27	0.314
**Anthropometric characteristics**			
Body weight trajectories 2002–2012, %			0.065
Never overweight/obese	11	20	
Became overweight	29	30	
Became non-overweight/obese	17	17	
Always overweight/obese	43	33	
**Lifestyle characteristics**			
Smoking trajectories (2002–2012), %			0.569
Never smoked	48	55	
Started smoking	11	9	
Stopped smoking	14	11	
Always smoked	27	25	
Physical activity trajectories (2002–2012), %			0.526
Always physically inactive	45	38	
Started physical activity	13	14	
Stopped physical activity	32	35	
Always physically active	9	13	
Mediterranean diet trajectories (2002–2012), %			0.394
Always close	15	20	
From close to away	50	49	
From away to close	15	18	
Always away	19	14	
MedDietScore (2002), Median (IQR)	26 (2)	27 (3)	**0.014**
MedDietScore (2012), Median (IQR)	25 (3)	26 (3.0)	**0.019**

* *p*-Values referring to differences between CVD status and categorical variables are derived from the chi-squared test. *p*-Values referring to differences between sleep habits and continuous variables are derived from Student’s *t*-test (i.e., age, normally distributed) or the Mann–Whitney non-parametric test (i.e., MedDietScore at 2002 and 2012, non-normally distributed). Bold indicates statistical significance. Abbreviations: CVD: cardiovascular disease; IQR: interquartile range; SD: standard deviation; SES: socioeconomic status.

**Table 3:** Baseline and longitudinal characteristics according to Mediterranean diet adherence and sleep habits of the study participants (*n* = 313) at the 10-year follow-up.

	MD Adherence and Sleep Habits				*p*-Value *
Baseline Socio-Demographic Characteristics	Low MD and Inadequate Sleep	Low MD and Adequate Sleep	High MD and Inadequate Sleep	High MD and Adequate Sleep	
*Ν*	99	53	81	80	-
Age, mean (SD)	48 (9)	47 (9)	41 (9) ^a,b^	38 (9) ^a,b^	**<0.001**
Sex, %men	75	66	41 ^a,b^	45 ^a,b^	**<0.001**
SES, %					**0.016**
Low	11	4	5	5	
Middle	49	38	61	35	
High	40	58	34	60	
**Baseline clinical characteristics**					
History of hypertension, %	39	32	18 ^a^	12 ^a,b^	**<0.001**
History of hypercholesterolemia, %	47	46	43	38	0.577
History of diabetes mellitus, %	9	3	1 ^a^	1 ^a^	**0.002**
Family history of CVD, %	49	48	43	46	0.481
**Clinical characteristics at 20 years**					
History of hypertension, %	71	57	39 ^a^	26 ^a,b^	**<0.001**
History of hypercholesterolemia, %	87	71	74	71	**0.035**
History of diabetes mellitus, %	36	30	28	25	0.398
**Anthropometric characteristics**					
Body weight trajectories 2002–2012, %			^a,b^	^a,b^	**<0.001**
Never overweight/obese	6	14	17	24	
Became overweight	12	18	49	37	
Became non-overweight/obese	25	23	7	13	
Always overweight/obese	57	45	27	26	
**Lifestyle characteristics**					
Smoking trajectories (2002–2012), %					0.159
Never smoked	41	50	57	57	
Started smoking	15	11	6	9	
Stopped smoking	17	14	10	9	
Always smoked	27	25	27	25	
Physical activity trajectories (2002–2012), %					0.154
Always physically inactive	49	43	40	35	
Started physical activity	10	6	18	19	
Stopped physical activity	34	37	30	34	
Always physically active	7	14	12	12	
Mediterranean diet trajectories (2002–2012), %			^a,b^	^a,b^	**<0.001**
Always close	0	0	33	33	
From close to away	50	54	50	46	
From away to close	14	12	17	21	
Always away	36	34	0	0	
MedDietScore (2002), Median (IQR)	25.6 (1.5)	25.7 (1.6)	27.2 (1.7) ^a,b^	27.4 (2.2) ^a,b^	**<0.001**
MedDietScore (2012), Median (IQR)	24.1 (3.8)	24.3 (5.8)	27 (2.0) ^a,b^	27.6 (2) ^a,b^	**<0.001**

* *p*-Values referring to differences between MD adherence and sleep habits and categorical variables are derived from the chi-squared test. *p*-Values referring to differences between MD adherence and sleep habits and continuous variables are derived from ANOVA (i.e., age, normally distributed) or the Kruskal–Wallis nonparametric test (i.e., MedDietScore at 2002 and 2012, non-normally distributed). Bold indicates statistical significance. ^a^ Post-hoc comparisons vs. low MD and inadequate sleep, performed using the Bonferonni rule (*p*-value < 0.05). ^b^ Post-hoc comparisons vs. low MD and adequate sleep, performed using the Bonferonni rule (*p*-value < 0.05). Adequate sleep is defined as ≥7 h/day, and inadequate sleep as <7 h/day. Low MD adherence was defined as MedDietScore during 2012 <27 (median), and high MD adherence as MedDietScore during 2012 ≥27 (median). Abbreviations: ANOVA: analysis of variance; CVD: cardiovascular disease; IQR: interquartile range; MD: Mediterranean diet; SD: standard deviation; SES: socioeconomic status.

**Table 4:** Results from Cox proportional hazards models evaluating the association between adherence to the Mediterranean-type diet and cardiovascular disease risk (*n* = 313).

Models	Adjustments	HR (95%CI) *
**Model 1**	-	**0.85 (0.83, 0.87)**
**Model 2**	Age, sex, SES	**0.93 (0.89, 0.96)**
**Model 3**	Model 2+ hypertension, hypercholesterolemia, diabetes, family history of CVD	**0.92 (0.88, 0.97)**
**Model 4**	Model 3+ trajectories of body weight, physical activity level, MD adherence, smoking habits	**0.91 (0.86, 0.97)**
**Model 5**	Model 4+ adequate sleep	0.88 (0.79, 1.00)

* Cells indicate hazard ratios and 95% confidence intervals taken from Cox proportional hazards models concerning the risk of cardiovascular disease per 1/55 increase of MedDietScore. Bold color indicates statistical significance. Abbreviations: CI: confidence interval; CVD: cardiovascular disease; HR: hazard ratios; SES: socioeconomic status.

**Table 5:** Subgroup analyses evaluating the relationship between Mediterranean-type diet adherence and cardiovascular disease risk stratified by adequate sleep habits (*n* = 313).

	MedDietScore, per 1/55 Increase *	Adjustments
Sleep Habits		Age, sex, SES, hypertension, hypercholesterolemia, diabetes, family history of CVD, trajectories of body weight, physical activity level, Mediterranean diet adherence, smoking habits
Inadequate sleep, <7 h/day	0.95 (0.82, 1.09)	
Adequate sleep, ≥7 h/day	**0.80 (0.65, 0.98)**	

* Cells indicate hazard ratios and 95% confidence intervals taken from Cox proportional hazards models concerning the risk of CVD per 1/55 increase of MedDietScore. Bold color indicates statistical significance. Abbreviations: CVD: cardiovascular disease; SES: socioeconomic status.

**Table 6:** Results from Cox proportional hazards models evaluating the association between the concomitant adherence to the Mediterranean type diet and sleep habits and cardiovascular disease risk (*n* = 313).

MD Adherence and Sleep Habits	HR (95%CI) *	Models	Adjustments
Low MD and inadequate sleep	Reference category	Model 1	-
Low MD and adequate sleep	0.77 (0.38, 1.48)		
High MD and inadequate sleep	**0.45 (0.24, 0.85)**		
High MD and adequate sleep	**0.14 (0.06, 0.32)**		
Low MD and inadequate sleep	Reference category	Model 2	age, sex, SES
Low MD and adequate sleep	1.45 (0.62, 3.34)		
High MD and inadequate sleep	0.68 (0.29, 1.57)		
High MD and adequate sleep	**0.30 (0.11, 0.82)**		
Low MD and inadequate sleep	Reference category	Model 3	2+ hypertension, hypercholesterolemia, diabetes, family history of CVD
Low MD and adequate sleep	1.10 (0.51, 2.38)		
High MD and inadequate sleep	0.74 (0.34, 1.58)		
High MD and adequate sleep	**0.25 (0.10, 0.64)**		
Low MD and inadequate sleep	Reference category	Model 4	3+ trajectories of body weight, physical activity level, MD adherence, smoking habits
Low MD and adequate sleep	1.31 (0.58, 2.96)		
High MD and inadequate sleep	0.90 (0.39, 2.06)		
High MD and adequate sleep	**0.30 (0.11, 0.80)**		

* Cells indicate hazard ratios and 95% confidence intervals taken from Cox proportional hazards models concerning the risk of CVD in each of the 4 groups concerning the concomitant adherence to the MD and sleep habits. Bold color indicates statistical significance. Adequate sleep is defined as ≥7 h/day, and inadequate sleep as <7 h/day. Low MD adherence was defined as MedDietScore during 2012 <27 (median), and high MD adherence as MedDietScore during 2012 ≥27 (median). Abbreviations: CI: confidence interval; CVD: cardiovascular disease; HR: hazard ratios; MD: Mediterranean diet; SES: socioeconomic status.

## Data Availability

The data presented in this study are available on request from the corresponding author. The data are not publicly available due to privacy restrictions.
